# Chemical Pretreatment Activated a Plastic State Amenable to Direct Lineage Reprogramming

**DOI:** 10.3389/fcell.2022.865038

**Published:** 2022-03-25

**Authors:** Zhenghao Yang, Xiaochan Xu, Chan Gu, Alexander Valentin Nielsen, Guokai Chen, Fan Guo, Chao Tang, Yang Zhao

**Affiliations:** ^1^ State Key Laboratory of Natural and Biomimetic Drugs, MOE Key Laboratory of Cell Proliferation and Differentiation, Beijing Key Laboratory of Cardiometabolic Molecular Medicine, Institute of Molecular Medicine, College of Future Technology, Peking University, Beijing, China; ^2^ Peking-Tsinghua Center for Life Sciences, Peking University, Beijing, China; ^3^ State Key Laboratory of Stem Cell and Reproductive Biology, Institute of Zoology, Chinese Academy of Sciences, Beijing, China; ^4^ The Niels Bohr Institute, University of Copenhagen, Copenhagen, Denmark; ^5^ Centre of Reproduction, Development and Aging, Faculty of Health Sciences, University of Macau, Macau, China; ^6^ Center for Quantitative Biology, Peking University, Beijing, China

**Keywords:** chemical reprogramming, cell plasticity, chromatin accessibility, cell fate transition, direct reprogramming

## Abstract

Somatic cells can be chemically reprogrammed into a pluripotent stem cell (CiPSC) state, mediated by an extraembryonic endoderm- (XEN-) like state. We found that the chemical cocktail applied in CiPSC generation initially activated a plastic state in mouse fibroblasts before transitioning into XEN-like cells. The plastic state was characterized by broadly activated expression of development-associated transcription factors (TFs), such as *Sox17*, *Ascl1*, *Tbx3*, and *Nkx6-1*, with a more accessible chromatin state indicating an enhanced capability of cell fate conversion. Intriguingly, introducing such a plastic state remarkably improved the efficiency of chemical reprogramming from fibroblasts to functional neuron-like cells with electrophysiological activity or beating skeletal muscles. Furthermore, the generation of chemically induced neuron-like cells or skeletal muscles from mouse fibroblasts was independent of the intermediate XEN-like state or the pluripotency state. In summary, our findings revealed a plastic chemically activated multi-lineage priming (CaMP) state at the onset of chemical reprogramming. This state enhanced the cells’ potential to adapt to other cell fates. It provides a general approach to empowering chemical reprogramming methods to obtain functional cell types bypassing inducing pluripotent stem cells.

## Introduction

Somatic cells can be chemically reprogrammed into functional cell types indirectly by first becoming pluripotent through a XEN-like state (Hou et al., 2013; Zhao et al., 2015) or directly without an intermediate pluripotent state. The application superiority of the chemical reprogramming strategy over the transgenic approach in inducing cell fate reprogramming is well established (Zhao, 2019). For example, small molecules are genetically non-integrative, easy to be manipulated, cell-culture standardized, and cost-effective. Chemical cocktails could also help increase efficiency in generating a defined cell type (Zhao et al., 2015). To date, chemical reprogramming has been a promising strategy for obtaining functional cell types in regenerative medicine. Fibroblasts were reported to be reprogrammed into many cell types, including neural progenitors (Cheng et al., 2014), neuron cells (Hu et al., 2015; Li et al., 2015; Mahato et al., 2020; Yin et al., 2019), cardiomyocytes (Fu et al., 2015; Cao et al., 2016), skeletal muscles (Bansal et al., 2019), brown adipocytes (Nie et al., 2017; Takeda et al., 2017), astrocytes (Tian et al., 2016), and endoderm progenitor-like cells (Cao et al., 2017; Wang et al., 2016).

However, the roles of chemicals in reprogramming systems are still elusive, which hampered the development of chemical cocktails for an assigned cell type. In the transgenic approach, the reprogramming factors are always the target cell type enriched TFs. Those TFs are associated with the target cell type’s development or differentiation. They have been intensively studied in somatic reprogramming into induced pluripotency stem cells (iPSCs) and direct lineage reprogramming (Takahashi and Yamanaka, 2006; Xu et al., 2015). They directly serve as the pioneer factors to shape specific cell type favored epigenetic states and activate the expression of other master TFs for cell fate reprogramming (Iwafuchi-Doi and Zaret, 2014). Unlike these reprogramming genes, the mechanisms of chemicals in reprogramming and determining a cell fate are far from known.

Notably, the small molecules essential for CiPSC induction, CHIR99021 (a GSK3 inhibitor), 616452 (Repsox, an ALK5 inhibitor), and Forskolin (a cAMP agonist) and their combinations have been frequently used for the direct induction of different cell types (Zhao, 2019). It suggests that some common mechanisms are underlying these chemical-induced cell-type reprogramming processes. Understanding the mechanisms is beneficial to developing additional chemical reprogramming systems based on the same rationale.

Herein, we found that mouse fibroblasts were initially induced into a plastic chemically activated multi-lineage priming (CaMP) phase in chemical reprogramming before further specification into specific lineages. The CaMP phase was characterized by heterogeneous expression of multiple developmental genes and a global gain of chromatin accessibility. It was induced concomitantly by core small molecules, CHIR99021, 616452, and Forskolin. Introducing the CaMP phase with a chemical pretreatment, we improved the chemical reprogramming systems from fibroblasts directly into functional neuron-like cells and beating myocytes.

## Materials and Methods

### Mice

All procedures involving mice were approved by the Institutional Animal Care and Use Committee (IACUC) and Use Committee at the Peking University, Beijing. For lineage-tracing experiments, 12–16 weeks Col1a2-CreERT2 and Rosa26tdTom mice were used. For *in vivo* labeling, all pregnant female mice have received intraperitoneal injections of tamoxifen (20 mg/ml, Sigma, United Kingdom) at a dose of 4 mg/30 g body weight before the isolation of MEF.

### MEF Isolation

MEF medium: high glucose Dulbecco’s modified Eagle’s medium (DMEM) supplemented with 10% fetal bovine serum (FBS), 1% GlutaMAX, 1% nonessential amino acids (NEAAs), 0.055 mM 2-mercaptoethanol, and 1% penicillin-streptomycin. Mouse embryonic fibroblasts (MEFs) were isolated from ICR mouse embryos. Briefly, after removal of the head, limbs, and viscera, E13.5 embryos were minced with scissors and dissociated in trypsin-EDTA at 37°C for 10 min. After adding MEF medium and centrifugation, MEF cells were collected and cultured.

### Generation of XEN-Like Cells From Fibroblasts

XEN reprogramming medium: KnockOut DMEM supplemented with 10% KnockOut Serum Replacement (KSR), 10% FBS, 1% GlutaMAX, 1% NEAAs, 0.055 mM 2-mercaptoethanol, 1% penicillin-streptomycin (Invitrogen), 50 ng/ml basic fibroblast growth factor (bFGF), and the small-molecule cocktail VC6FAE (0.5 mM valproic acid, 20 μM CHIR99021, 10 μM 616,452, 50 μM Forskolin, 0.05 μM AM580, and 5 μM EPZ004777). MEFs were seeded at 20,000 cells per well of a 12-well plate with an MEF culture medium. For XEN induction, the medium was changed to XEN reprogramming medium the next day, and it was changed every 4 days for 12–20 days.

### Induction of Skeletal Muscle Cells From CaMP State

Skeletal muscle reprogramming medium: DMEM/M199 medium (4:1) supplemented with 10% KSR, 10% FBS, 1% GlutaMAX, 1% NEAAs, 1% penicillin-streptomycin (Invitrogen), and the small-molecule cocktail C6FS (20 μM CHIR99021, 10 μM 616,452, 50 μM Forskolin, and 3 μM SB431542). MEFs were seeded at 20,000 cells per well of a 12-well plate with an MEF culture medium. For skeletal muscle cell induction, the medium was changed to XEN reprogramming medium the next day (day 0), and the medium was switched to skeletal muscle reprogramming medium at day 4 to induce myocytes for 8–12 days. The skeletal muscle reprogramming medium was changed every 4 days.

### Induction of Neuron-Like Cells From CaMP State

Neuron-like cells reprogramming medium: neurobasal plus medium supplemented with 2% B27-plus supplement, 1% GlutaMAX, 1% penicillin-streptomycin (Invitrogen), and the small-molecule cocktail CFI (3 μM CHIR99021, 10 μM Forskolin, and 10 μM ISX-9).

MEFs were seeded at 20,000 cells per well of a 12-well plate with an MEF culture medium. For neuron-like cells induction, the medium was changed to XEN reprogramming medium the next day (day 0), and the medium was switched to neuron-like cells reprogramming medium at day 4 to induce neuron-like cells for 8–12 days. For neuron-like cells maturation, cells were plated on astrocytes at day 12 or day 16, and further culture for 16 days was supplemented with BDNF (20 ng/ml) and GDNF (20 ng/ml). Neuron-like cells reprogramming medium was changed every 4 days.

### Isolation of Astrocytes

Astrocyte medium: DMEM/F12 supplemented with 10% FBS, 1% GlutaMAX, 1% nonessential amino-acids (NEAAs), and 1% penicillin-streptomycin.

After the newborn mice were anesthetized on ice and sacrificed, they were disinfected with 75% alcohol for 5 min. Brain tissue was taken and cut into pieces of 4 mm^3^ with scissors. Pieces of brain tissue were collected and digested with 2 ml 0.25% trypsin and 0.1 ml DNase I (2 mg/ml) for 20 min at 37°C. The digestion was stopped with 2 ml astrocyte medium and centrifuged for 5 min at 1,500 rpm. About 600,000 cells were resuspended with 10 ml astrocyte medium and plated into 10 cm dish. The supernatant was taken into a new T75 flask after 30 min and cultured for 7–10 days. After that, cultured cells were shaken on a shaker for 16 h at 250 rpm. Adherent astrocytes were digested and plated for neuron-like cells maturation.

### Isolation of Neuron Cells

Neuron culture medium: neurobasal plus medium supplemented with 2% B27-plus supplement, 1% GlutaMAX, 1% penicillin-streptomycin. The plating medium was prepared with a neuron culture medium supplement with 10% FBS.

After the newborn mice were anesthetized on ice and sacrificed, they were disinfected with 75% alcohol for 5 min. Brain tissue was taken and cut into pieces of 9 mm^3^ with scissors. Pieces of brain tissue were collected and digested with 3 ml 0.1% trypsin and 0.1 ml DNase I (2 mg/ml) for 9 min at 37 °C. We discarded the supernatant and add 4 ml plating medium. After that, we discarded the supernatant, added 1.5 ml plating medium and 0.1 ml DNase I (2 mg/ml) and pipette 20 times, and collected the supernatant. We repeated this step one more time and centrifuged the collected supernatant for 5 min at 1,000 rpm. Cells were seeded at 25,000 cells per well of a poly-l-lysine pre-coated 12-well plate with a plating medium. We then gently shook the 12-well plate and switched the medium to neuron culture medium 6 h later, and cells were cultured at 37°C.

### Isolation and Culture of Myocytes

After the newborn mice were anesthetized on ice and sacrificed, they were disinfected with 75% alcohol for 5 min. Limb tissue was taken and cut into pieces of 0.1 mm^3^ with scissors. Pieces of limb tissue were collected and digested with 6 ml 0.25% trypsin and 0.2 ml DNase I (2 mg/ml) for 20 min at 37°C, pipette tissue every 5 min. Cells were centrifuged for 10 min at 1,000 rpm and 500,000 cells were plated into 10 cm dish for 2 h. Cells in the supernatant were transferred into a new 10 cm dish for further culture. After 3 days, the medium was switched into skeletal muscle differentiation medium (DMEM medium supplement 2% horse serum) for further culture.

### Immunofluorescence

Cells were washed with PBS and fixed in 4% paraformaldehyde for 15 min at room temperature. After washing twice with PBS, cells were permeabilized and blocked in PBS containing 0.2% Triton X-100 and 3% donkey serum for 1 h at room temperature. Then, the cells were incubated with primary antibodies at 4°C overnight. After washing three times with PBS, secondary antibodies (Jackson ImmunoResearch) were incubated at 37°C for 1 h. The nuclei were stained with DAPI (Roche Life Science) for 5 min. Primary antibodies were those specific to rabbit anti-SALL4 (Abcam, 1:500), mouse anti-Tubb3 (Biolegend, 1:300), rabbit anti-Synapsin 1 (Abcam, 1:500), rabbit anti-Map2 (Millipore, 1:200), rabbit anti-Neun (Millipore 1:500), rabbit anti-GABA (Sigma, 1:300), anti-mouse neurofilament 200 (Millipore 1:300), rabbit anti-vGlut1 (Synaptic system, 1:300), mouse anti-myosin heavy chain (R&D, 1:300), mouse anti-MyoD (Thermo fisher, 1:200), mouse anti-myogenin (Thermo fisher, 1:200), and mouse anti-α-actinin (Sigma, 1:500). The secondary antibodies used were FITC-conjugated secondary antibodies and TRITC-conjugated secondary antibodies (Jackson ImmunoResearch, 1:200).

### RT-qPCR

Total RNA was extracted using the EasyPure RNA Kit (TransGen Biotech) and was reverse-transcribed into cDNA using TransScript One-step gDNA Removal and cDNA Synthesis SuperMix (TransGen Biotech). Real-time PCR was performed on a Quantagene q225 System (KUBO technology) using 2×T5 Fast qPCR Mix (TSINGKE Biological Technology).

### ATAC-Seq

ATAC-seq libraries were prepared using Trueprep DNA library Prep Kit V2 for Illumina (vazyme). Totally, 50,000 cells were used for every single reaction. Cells were washed in 100 μl cold PBS and resuspended in 50 μl lysis buffer (10 mM Tris-HCl pH 7.4, 10 mM NaCl, 3 mM MgCl_2_, 0.5% NP-40) for 10 min, and nuclei were spun at 500 g for 10 min using a refrigerated centrifuge. Then, the pellet was resuspended in 50 μl transposase reaction mix and incubated at 37°C for 30 min. The samples were purified and purified, and then libraries were amplified by PCR for 13 cycles. The libraries were sequenced using an Illumina HiSeq 2,500 machine.

### RNA-Seq

We treated MEF cells with C6FAE, 6FAE, CFAE, or C6AE from day 0 and extracted total RNA on day 4, day 8, and day 12. Total RNA was extracted using the EasyPure RNA Kit (TransGen Biotech). Library construction was completed by Novogene company. The libraries were sequenced using an Illumina HiSeq 2,500 machine.

### Electrophysiology

Whole-cell patch-clamp recordings were performed by Scope Research Institute of Electrophysiology. All currents were recorded using a MultiClamp 700 A amplifier. For whole-cell patch-clamp recordings, the ACSF (artificial cerebrospinal fluid) extracellular solution contained 128 mM NaCl, 30 mM glucose, 25 mM HEPES, 5 mM KCl, 2 mM Ca^2+^, and 1 mM MgCl_2_. The pH of the bath solution was adjusted to 7.3 with NaOH, and osmolality was 300–305 mOsm/L. The pipette solution consisted of 135 mM KCl, 5 mM Na-phosphocreatine, 10 mM HEPES, 2 mM EGTA, 4 mM Mg-ATP, and 0.5 mM Na_2_-GTP. The pH of pipette solution was adjusted to 7.3 with KOH and osmolality to 280–290 mOsm/L. Whole-cell patch-clamp recordings were carried out using a HEKA EPC10 amplifier with PatchMaster software (HEKA; Instrument Inc., Lambrecht/Pfalz, Germany). To record the sodium and potassium currents, cells were held at −80 mV and depolarized from −80 to +80 mV in 10 mV increments for 1 s. The sample and sweep intervals were 20 µs and 2 s, respectively. To record spontaneous excitatory postsynaptic currents (EPSCs), induced neuron-like cells were held at −70 and 0 mV, respectively.

### Data Processing and Analysis

The data used in the RNA-Seq analysis was combined with our previous data (Yang et al., 2020), and we additionally performed RNA-Seq on the C6F5UE treatment group. Gene expression levels were normalized as log_2_ (FPKM+1) in all bulk RNA-Seq data. For temporal bulk RNA-Seq data clustering in each cocktail treatment, genes that have detected FPKM >1 at least at one sample remained to perform *K*-means clustering and were grouped into 20 clusters. For the cell fate induction experiment, genes that vary >1 among all samples remained to perform heterarchical clustering. PCA was done with all normalized gene expression levels with scaling the normalized expression for each gene by *z*-score among samples. Day 16 and XEN data were adapted from GEO Datasets (GEO IDs: GSE73631). For dropout experiments, gene expression under C6FAE conditions was first compared in MEFs on day 4 and day 8. TFs that increased at least by 1.5 were identified as upregulated and retained for the follow-up analysis.

Single-cell data was adapted from the previously published dataset (GEO IDs: GSE144097). We used Seurat (Stuart et al., 2019) package to do t-distributed stochastic neighbor embedding (t-SNE) projection and visualization. The three germ layer transcriptional factors correlation was calculated with the spearman correlation coefficients between gene pairs with log_2_(UMI count +1) and visualized with heatmap.2 function from ggplot2 package.

ATACseq analysis includes peak calling with MACS (version 2.1.2) (Zhang et al., 2008), differential peak detection with RPKM, and visualization with EnrichedHeatmap (Gu et al., 2018). Two biological replications of Control and CaMP samples were processed and CaMP enriched peak regions and Control enriched peak regions were labeled. Coverage tracks of the samples were generated with the alignment of reads (BAM file) with the bamCoverage function from deepTools (Ramirez et al., 2014). The number of reads per bin was calculated and normalized by reads per kilobase per million mapped reads (RPKM).

These analyses were done with MATLAB and R script.

## Results

### Chemical Reprogramming Cocktails Initially Activated the Expression of a Broad Spectrum of Development-Associated Transcription Factors

**FIGURE 1:**
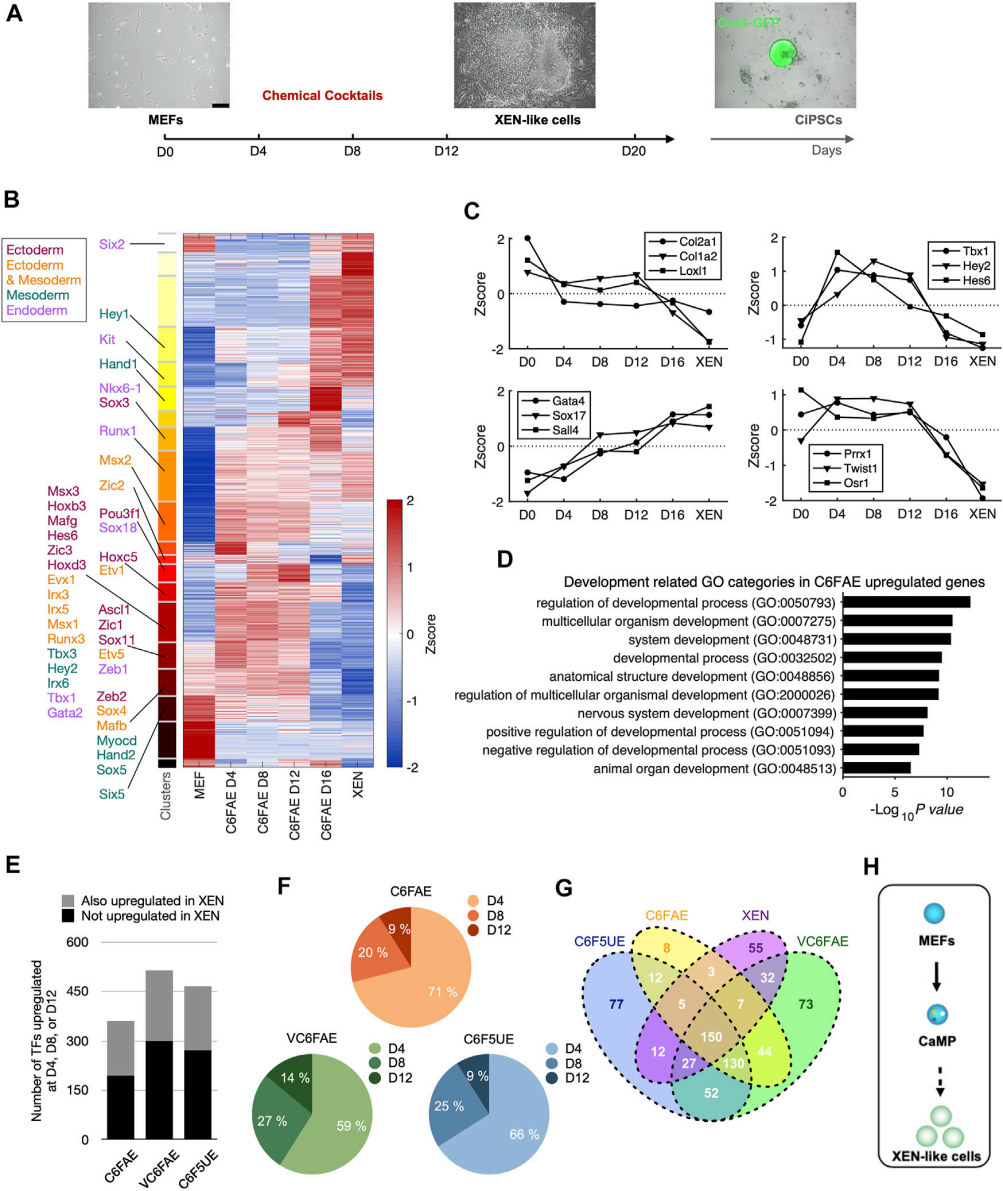
Chemical cocktails activate the endogenous expression of transcription factors for multiple lineages. **(A)** Schematic diagram of chemical reprogramming and bulk RNA-Seq sampling. **(B)** Clustering of temporal gene expression dynamics in the early stage of chemical reprogramming. *K*-means clustering partitioned genes which FPKM >1 at least one-time point into 20 clusters. Known transcription factors (TFs) governing three germ layers development are labeled to the corresponding clusters. **(C)** Representative gene expression dynamics during the early stage of chemical reprogramming. Left top: continuous decline; right top: increase and decline; left bottom: continuous increase; right bottom: late decline. **(D)** Chemical cocktail, C6FAE, upregulated genes in development-associated GO categories. Gene ontology analysis was performed with genes that expressed at least 1.5 larger than MEF in normalized measurement (log_2_(FPKM+1)) at day 4, day 8, or day 12. **(E)** Number of upregulated TFs with different chemical cocktail treatments. Height of bars: total numbers; grey: TFs upregulated in the first 12 days and in XEN cell type; black: TFs upregulated in the first 12 days but not in XEN cell type. **(F)** Percentages of upregulated TFs at different time points with different cocktail treatments. TFs were aligned to the time points based on the first time they show 1.5 larger than MEF in normalized expression. **(G)** Venn diagram of upregulated TFs within different cocktail treatments (day 4, 8, or 12) and XEN cells compared with MEF. **(H)** Two steps schematic highlighting CaMP state in the early stage of XEN induction.

To investigate how chemical compounds alter cell fate, we previously studied the chemical reprogramming process from mouse embryonic fibroblasts (MEFs) to XEN-like cells. The study revealed a hierarchical activation of XEN cell master TFs primed by Sox17 and the different roles of essential chemicals during the process (Yang et al., 2020). In parallel to that study, we measured the global gene expression profiles by RNA sequencing during reprogramming. We treated the initial MEFs with chemical cocktails composed of CHIR99021 (C), 616452 (6), Forskolin (F), AM580 (A), and EPZ00477 (E) and collected the samples at days 0, 4, 8, and 12 (Figure 1A).

The gene expression induced by C6FAE showed various dynamics (Figure 1B). The genes were categorized into four major groups stepwise (Figures 1B,C): downregulated fibroblast genes in the first 4 days, upregulated genes from day 4 to day 12, upregulated XEN genes, and decreased master genes of fibroblast in the last period. Unexpectedly, those upregulated genes during fibroblast reprogramming to XEN-like cells included a broad spectrum of lineage-associated TFs, such as *Ascl1*, *Zic1*, *Hand2*, *Hey2*, *Nkx6-1*, and *Gata2*, which, respectively, regulate the development of ectoderm, mesoderm, and endoderm (Figures 1B,C). The top-ranked Gene Ontology terms of these lineage-associated genes included “regulation of developmental process,” “multicellular organismal development,” and “system development” (Figure 1D).

We also detected the activation of lineage-associated TFs in a chemical reprogramming system with additional chemical boosters for XEN generation, VPA, UNC0638 (Hou et al., 2013), and CH55 (Zhao et al., 2018) (Supplementary Figure S1). These different chemical cocktails activated lineage-associated TFs before XEN cell fate transition (Figure 1E). The activation timing for 56%–71% of these TFs can be as early as day 4 (Figure 1F). Interestingly, these upregulated lineage-associated TFs were highly overlapping in different chemical cocktails (Figure 1G). Thus, we refer to the induction phase with the activation of multi-lineage TFs, including Sox17 for XEN-like cell induction (Yang et al., 2020), as chemically activated multi-lineage priming (CaMP).

The XEN master genes *Sall4* and *Gata4* were significantly activated in the latest stage after the CaMP state (Figure 1C). It indicates that XEN cell fate was induced in a “plasticization and specification” manner rather than determined in the initial stage of chemical reprogramming (Figure 1H).

### Heterogeneous Expression of Endogenous Development-Associated Transcription Factors in Single Cells

The upregulated developmental genes could be activated in 1 cell simultaneously or in different cells heterogeneously. To clarify these two scenarios, gene expression in individual cells needs to be investigated. Thus, we re-analyzed our single-cell RNA-sequencing data obtained with SMART-seq2 (Yang et al., 2020).

**FIGURE 2:**
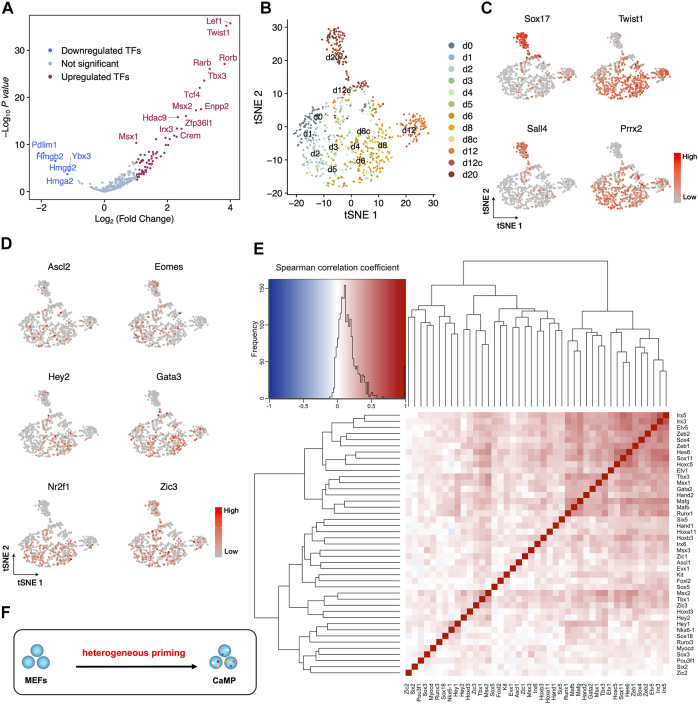
Heterogeneous expression of endogenous development-associated transcription factors in the CaMP phase. **(A)** Consistent with bulk RNA-Seq, upregulated TFs significantly increased expression in cells during the first 12 days of C6FAE induction compared with d0 (MEF) measured by single-cell RNA-Seq. The difference of expression for each gene is calculated as the difference of mean log_2_(UMI counts+1). *p*-value is calculated with two samples unpaired *t*-test. **(B)** t-SNE projection of single cells with upregulated TFs list from bulk RNA-Seq during the XEN reprogramming process. d8c and d12c, cells picked from colonies of day 8 (d8c) and day 12 (d12c). **(C)** Expression of XEN master genes (*Sox17*, *Sall4*) and MEF master genes (*Twist1, Prrx2*) in the t-SNE projection. **(D)** Examples of gene expression of activated development-associated TFs in individual cells on the t-SNE projection. **(E)** Spearman correlation between each pair of the three germ layer master genes. Left top: in the color key, color indicates the value (blue to red: −1 to 1), and histogram (black curve) indicates the statistic of Spearman correlation coefficients in the heatmap. **(F)** Schematic diagram of stochastic activation of endogenous TFs in the CaMP state.

We first confirmed that the upregulated TFs expression profiles are consistent in bulk RNA-Seq and single-cell RNA-Seq (Figure 2A). Cells from early induction time points (d0–d8) were mixed on the dimensional reduction projection by t-distributed stochastic neighbor embedding (t-SNE) with upregulated TFs detected from bulk RNA-Seq (Figure 2B). Cells harvested at day 20 (d20) stretched out of the major population and highly expressed XEN lineage marker genes, *Sox17* and *Sall4,* indicating successfully adapted XEN cell fate (Figure 2C). Cells harvested at day 12 (d12) close to the main group kept the expression of MEF master genes (*Twist1* and *Prrx2*) and a low level of *Sox17* (Figure 2C). This result denied the hypothesis that upregulated TFs were expressed in a group manner while supporting the other one together with the scatter pattern of their expression on the t-SNE map (Figure 2D). Thus, lineage-specific genes upregulated their mRNA levels in the early days heterogeneously.

Furthermore, correlation analysis shows that the expression of three germ layer master TFs also does not cluster the genes into groups (Figure 2E). The pairs of these TFs have low Spearman correlation coefficients except that a few pairs have slightly high coefficients around 0.5, such as Irx3 and Irx5, Mafb and Mafg, and Tbx3 and Msx1 (Figure 2F). This may be due to their inherent co-expression patterns or regulation relationships during development (Reinke et al., 2010; Gaborit et al., 2012; Munshi, 2012). Thus, the endogenous expression of TFs for multiple lineages was activated more stochastically. Therefore, the early initiated plastic state was formatted as heterogeneous priming (Figure 2F).

### Chemicals Induced a Global Gain of Chromatin Accessibility at CaMP State as Early as Day 4

We further explored the chromatin accessibility change of the CaMP state by ATAC-sequencing (ATAC-seq). We detected elevation in chromatin accessibility of the upregulated TFs after 4 days of treatment with chemical compounds.

**FIGURE 3:**
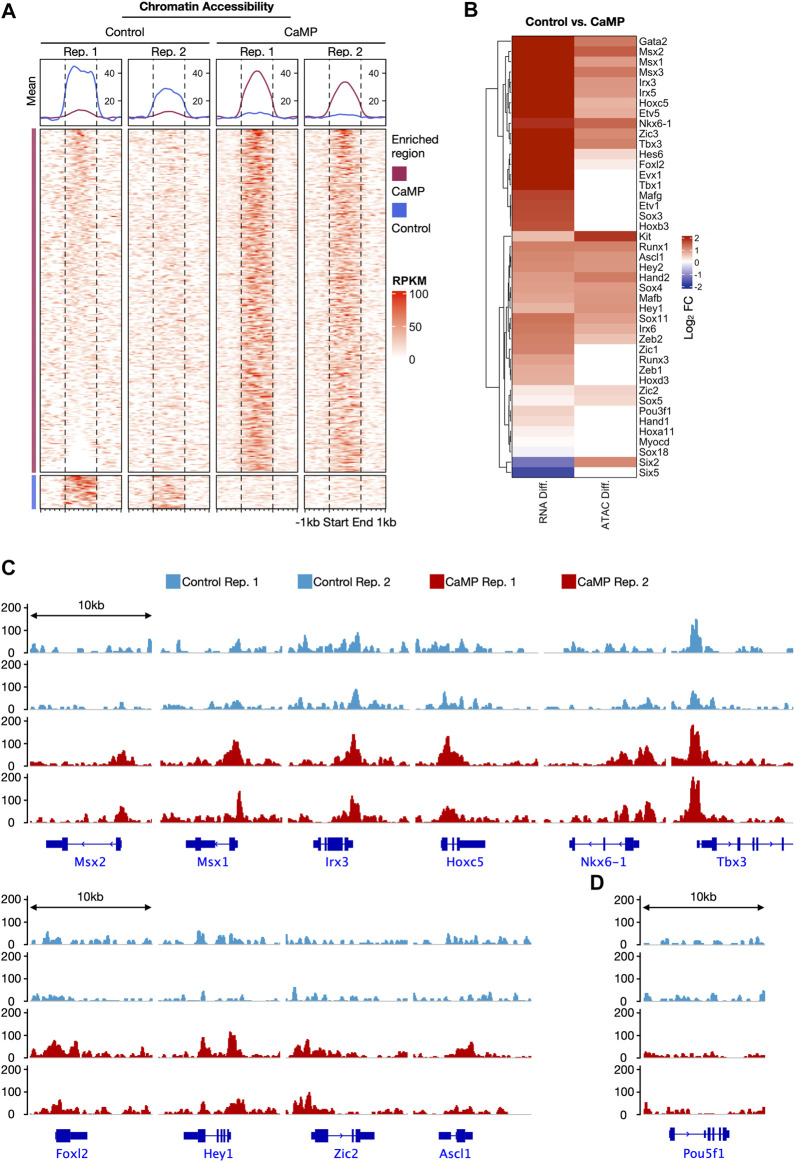
Chemicals induced a global gain of chromatin accessibility at CaMP phase as early as day 4. **(A)** Comparison of chromatin accessibility signals detected by ATAC-seq between Control and CaMP samples at day 4. Differential peak regions are shown with ±1 kb extension. The peak region signals were normalized with RPKM and clustered into “Control enriched region” and “CaMP enriched region.” The above panels show the mean RPKM value of corresponding columns and clusters. Both Control and CaMP conditions have two biological repeats. **(B)** Gene expression changes (RNA Diff.) and chromatin accessibility level changes (ATAC Diff.) of three germ layer master genes after 4 days of treatment of C6FAE compared with MEF. Different levels are quantified with the difference of log_2_(FPKM+1) and log_2_ (RPKM+1), respectively. **(C)** ATAC signal visualization of representative activated TFs with enhanced chromatin accessibility. RPKM range shown is scaled to the same among the four samples. **(D)** ATAC signal visualization of the pluripotency marker gene, *Pou5f1*. RPKM range shown is scaled to the same among the four samples.

Interestingly, we found that the CaMP phase significantly opened chromatin accessibility for a large number of genes compared to Control, and only a small proportion of genes had their chromatin accessibility state closed (Figure 3A). This finding was consistent with a substantial upregulation of gene expression induced by chemical compounds. The upregulated level of the activated three germ layer TFs in expression detected by RNA-Seq was positively correlated with the chromatin accessibility change (Figure 3B). In particular, some of the activated lineage-specific TFs, such as *Msx1*, *Nkx6-1*, *Tbx3*, *Zic2*, and *Ascl1*, had strong upregulation of chromatin accessibility after C6FAE treatment (Figure 3C). A few TFs in this list do not show a significant increase in chromatin accessibility due to their original highly open chromatin state (Supplementary Figure S2).

Moreover, chromatin accessibility of pluripotent genes *Pou5f1* was still not opened after 4 days of treatment with C6FAE (Figure 3D), which meant the global open of chromatin accessibility was not caused by the activation of the pluripotent gene. Overall, the change of chromatin accessibility was further in line with the activation of the development-associated TFs in the CaMP state.

### Core Reprogramming Chemicals (C6F) Concomitantly Induce the CaMP Phase

**FIGURE 4:**
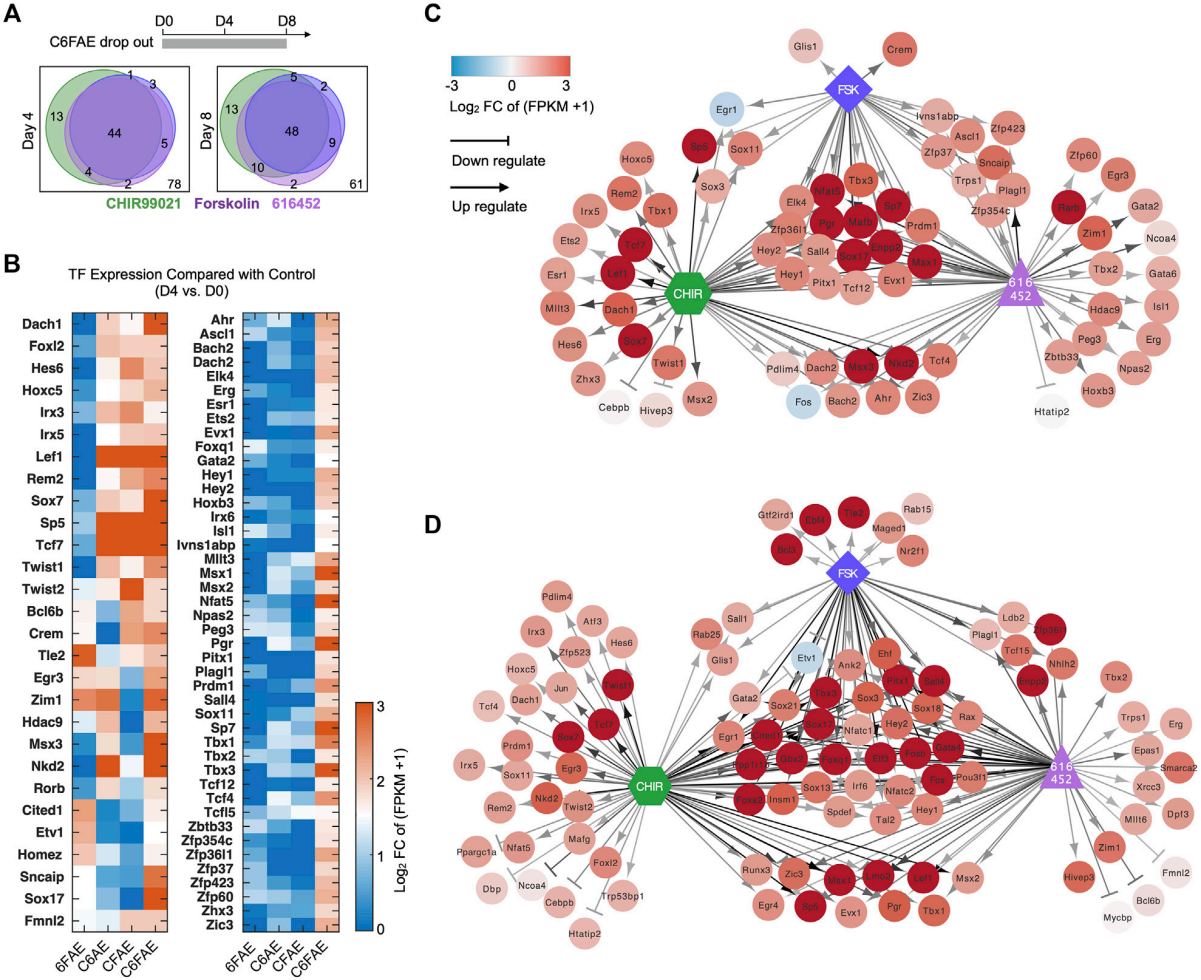
Core reprogramming chemicals (C6F) concomitantly induce the CaMP phase. **(A)** Venn diagram of TFs activated by C, 6, and F, respectively. The gene list was limited to 150 TFs activated by C6FAE. Genes were assumed to be activated by C, 6, or F at day 4 (or day 8) when they were upregulated more than 1.5 in normalized FPKM with the treatment of C6FAE while not being with subtracted each chemical. **(B)** TFs regulated by C, 6, and F during the first 4 days of XEN reprogramming. The expression of these TFs on day 4 was compared with their expression in MEFs in normalized FPKM. The color key is proportionate to the value of data of day 4 subtracted by data of MEFs. **(C, D)** TFs expressed differently when C, 6, and F were removed from the cocktail C6FAE in the first 4 days **(C)** and 8 days **(D)**. Red points: upregulated genes in C6FAE treated cells; blue points: downregulated genes in C6FAE treated cells. Arrows represent promotion, and horizontal lines represent inhibition. The darker lines mean stronger regulation.

We further investigated the roles of the essential reprogramming chemicals, CHIR99021, 616452, and Forskolin (C6F) in inducing the plastic CaMP phase. We compared the bulk RNA expression profiles of samples with the treatment of partial cocktails and those with full cocktails. CHIR99021, 616452, and Forskolin were essential for the transcriptional activation of most lineage-specific TFs in the CaMP state. Most of the TFs activated by the cocktail of C6FAE could not be activated without any one of CHIR99021, 616452, and Forskolin (Figures 4A,B). Subtracting any one of CHIR99021, 616452, and Forskolin from day 0 also hampered the expression of the XEN master TFs (Figures 4C,D). Thus, The cooperation of the three core chemicals activated the expression of those TFs in the CaMP phase.

In summary, CHIR99021, 616452, and Forskolin concomitantly initiated the CaMP state. All of them contributed to transcriptional activation of development-associated TFs, which explained why most previous chemical reprogramming systems used these three small molecules or those targeting the same pathways.

### Direct Chemical Reprogramming System Into Neuronal Lineage Was Empowered With CaMP Induction

Inspired by the molecular frameworks during cell fate specification in another study of us (Yang et al., 2020), the endogenously activated TFs of multiple lineages in the CaMP induction might be beneficial to induce cell types of other lineages. We found that the TFs of neurons, including Ascl1, a master gene for neuronal reprogramming, were also activated in the CaMP phase. It is possible to induce the neuron-like cells after CaMP induction more efficiently.

**FIGURE 5:**
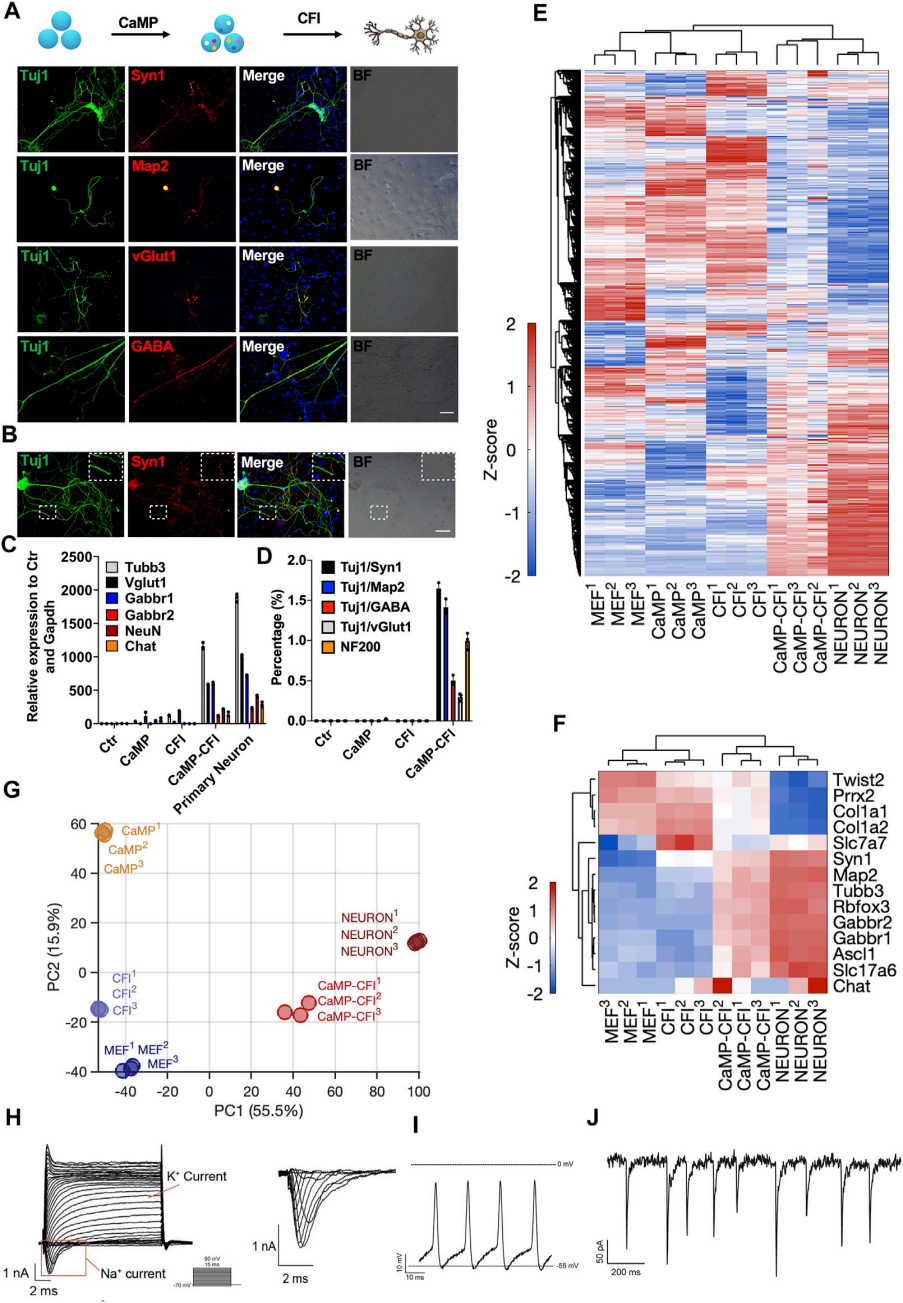
Direct chemical reprogramming system into neuronal lineage was empowered with CaMP induction. **(A)** Immunostaining for the chemically induced neuron-like cells with the CaMP pretreatment. Scale bar, 100 μm; CFI, CHIR99021/616452/Forskolin/ISX-9. **(B)** Immunostaining of CaMP-CFI induced functional synapses identified by Syn1 immunostaining. Scale bar, 100 μm. **(C)** Relative mRNA expression of typical neuronal marker genes induced with or without CaMP pretreatment. **(D)** The efficiency statistics of matured neuron-like cells identified by immunostaining. **(E)** Gene expression heatmap of all differentially expressed genes (normalized FPKM changed more than 1.5 among the samples) in neuron-like cells induced with or without CaMP step. The color key, Z-score of normalized FPKM. **(F)** Gene expression heatmap of typical neuron genes in neuron-like cells induced with or without CaMP step. The color key, Z-score of normalized FPKM. **(G)** PCA projection of neuron-like cells reprogramming processes analyzed with all differently expressed genes. The coefficient of variation (CV) was calculated for each gene among samples, and the PCA was performed with genes with CV larger than 0.1. **(H)** Action potentials of CaMP-CFI induced neuron-like cells after co-culture with astrocytes. One exemplary action potential trace was highlighted. **(I)** Spontaneous excitement potential of CaMP-CFI induced neuron-like cells after co-culture with astrocytes. **(J)** Spontaneous excitatory postsynaptic currents of CaMP-CFI induced neuron-like cells after co-culture with astrocytes.

By initially introducing CaMP state and changing the culture medium to which favored neuronal maintenance in culture and fine-tuning the composition of chemical cocktails after the CaMP phase, we found that a cocktail, CHIR99021, Forskolin, and ISX9 (CFI), drastically induced the transition from the CaMP to neuron-like cells only after the pretreatment of C6FAE for 4 days. The resulting induced neuron-like cells had more classic neuronal cell morphology and expressed typical neuronal markers Tuj1, Map2, Syn1, neuronfilam 200, and functional markers vGlut1, GABA, *Rbfox3*, *Gabbr2*, and *Chat* (Figures 5A–C). The efficiency of matured neuron-like cells identified by functional synapses marker-Syn1 and Tuj1 co-staining was about 1.7%, and the proportions of GABA or vGlut1 positive cells were about 0.5% and 0.3%, respectively (Figure 5D). In comparison, cells induced without the CaMP pretreatment expressed nearly no mature neural markers after 28 days of induction (Figure 5D).

By RNA sequencing, we found that neuron-like cells induced through CaMP had activated the expression of neuron-specific genes and similar expression profiles to primary neurons. (Figures 5E,F). By the principal component analysis, we found that the neuron-like cells induced through CaMP induction had transcriptional states closer to primary functional cells than cells induced without CaMP priming (Figure 5G). Importantly, action potential, spontaneous excitement potential, and spontaneous excitatory postsynaptic currents (EPSCs) were recorded in induced neuron-like cells after co-culture with astrocytes (Figures 5H–J and Supplementary Table S1).

### Direct Chemical Reprogramming System Into Myocytes Was Empowered With CaMP Induction

**FIGURE 6:**
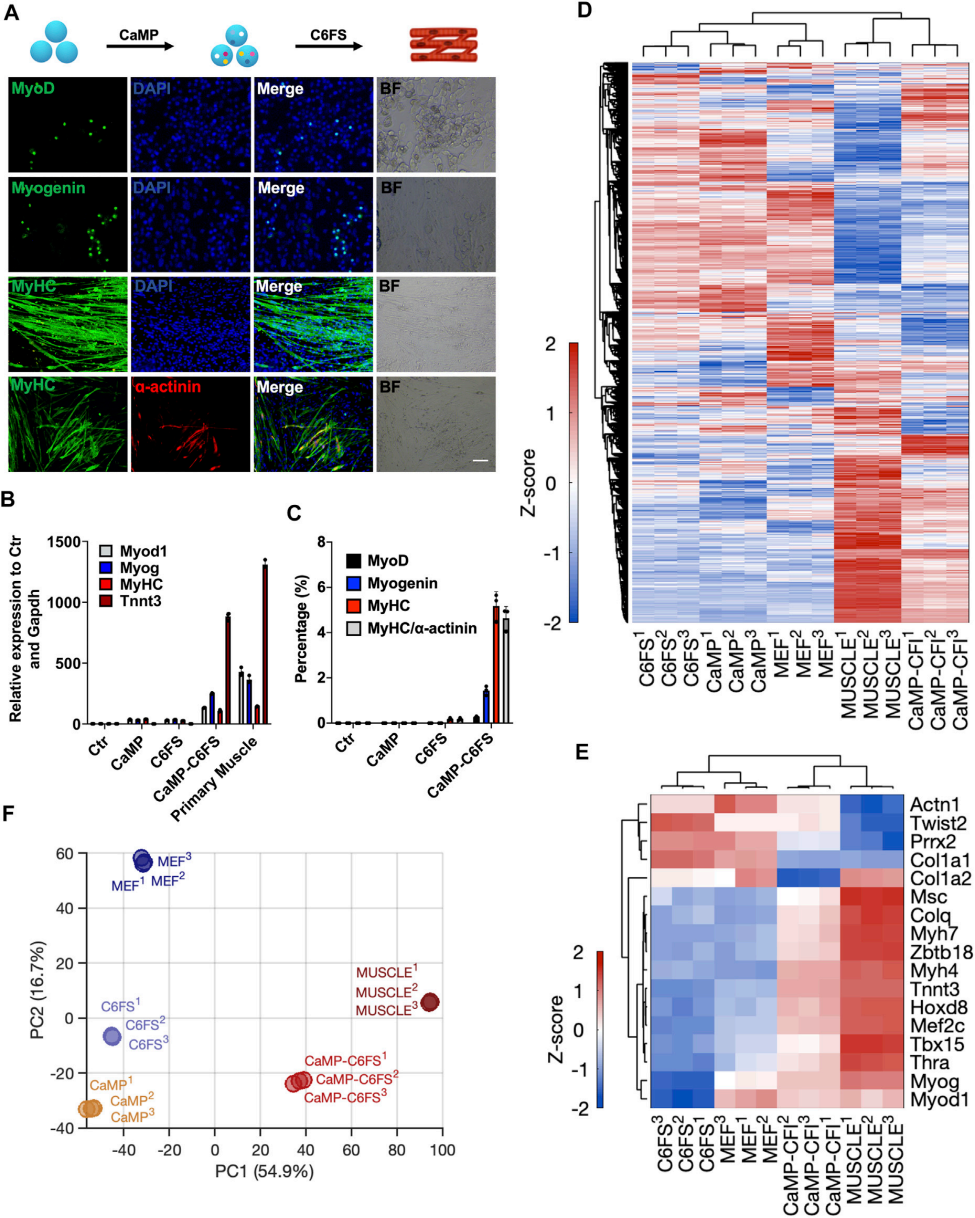
Direct chemical reprogramming system into myocytes was empowered with CaMP induction. **(A)** Induction of skeletal muscle cells with the CaMP pretreatment. Scale bar, 100 μm; C6FS, CHIR99021/616452/Forskolin/SB431542. **(B)** Relative mRNA expression of typical skeletal muscle marker genes induced with or without CaMP pretreatment. **(C)** The efficiency statistics of induced skeletal muscle cells identified by immunostaining. **(D)** Gene expression heatmap of all differentially expressed genes (normalized FPKM changed more than 1.5 among the samples) in skeletal muscle cells induced with or without CaMP step (analyzed by RNA-Seq of more than 30 clusters of induced skeletal muscle cells). The color key, Z-score of normalized FPKM. **(E)** Gene expression heatmap of typical skeletal muscle genes in skeletal muscle cells induced with or without CaMP step (analyzed by RNA-Seq of more than 30 clusters of induced skeletal muscle cells). The color key, Z-score of normalized FPKM. **(F)** PCA projection of myocytes reprogramming processes analyzed with all differently expressed genes. The coefficient of variation (CV) was calculated for each gene among samples, and the PCA was performed with genes with CV larger than 0.1.

Similarly, we optimized the myocytes’ induction through the CaMP state. By treating cells in the CaMP phase with myocyte culture medium containing CHIR99021, 616452, Forskolin, and SB431542 (C6FS), we induced contractile and multinucleated skeletal muscle cells expressing MyHC, Myog, Myod1, ɑ-actinin, and *Tnnt3* in 8–12 days, more efficient and faster than induction with only C6FS (Figures 6A,B and Supplementary video S1). The efficiency of MyHC and ɑ-actinin double-positive skeletal muscle cells was over 4% with P2 MEFs as starting cells. The efficiency of MyHC and ɑ-actinin double-positive skeletal muscle cells was over 4%, and the efficiency could reach 30% with P1 MEFs as starting cells. In comparison, few skeletal muscles cells could be induced without CaMP induction or specification stage with myocyte medium (Figure 6C).

By RNA sequencing, we found that the skeletal muscle cells induced through CaMP had activated the expression of myocyte-specific genes (Figures 6D,E). By principal component analysis, we found that the myocytes induced through CaMP induction had more comparable transcriptional profiles to primary functional cells than the cells induced without the CaMP priming (Figure 6F).

### Cells Induced From the CaMP State Are Not Derived From Progenitor Cells or Pluripotent Stem Cells

**FIGURE 7:**
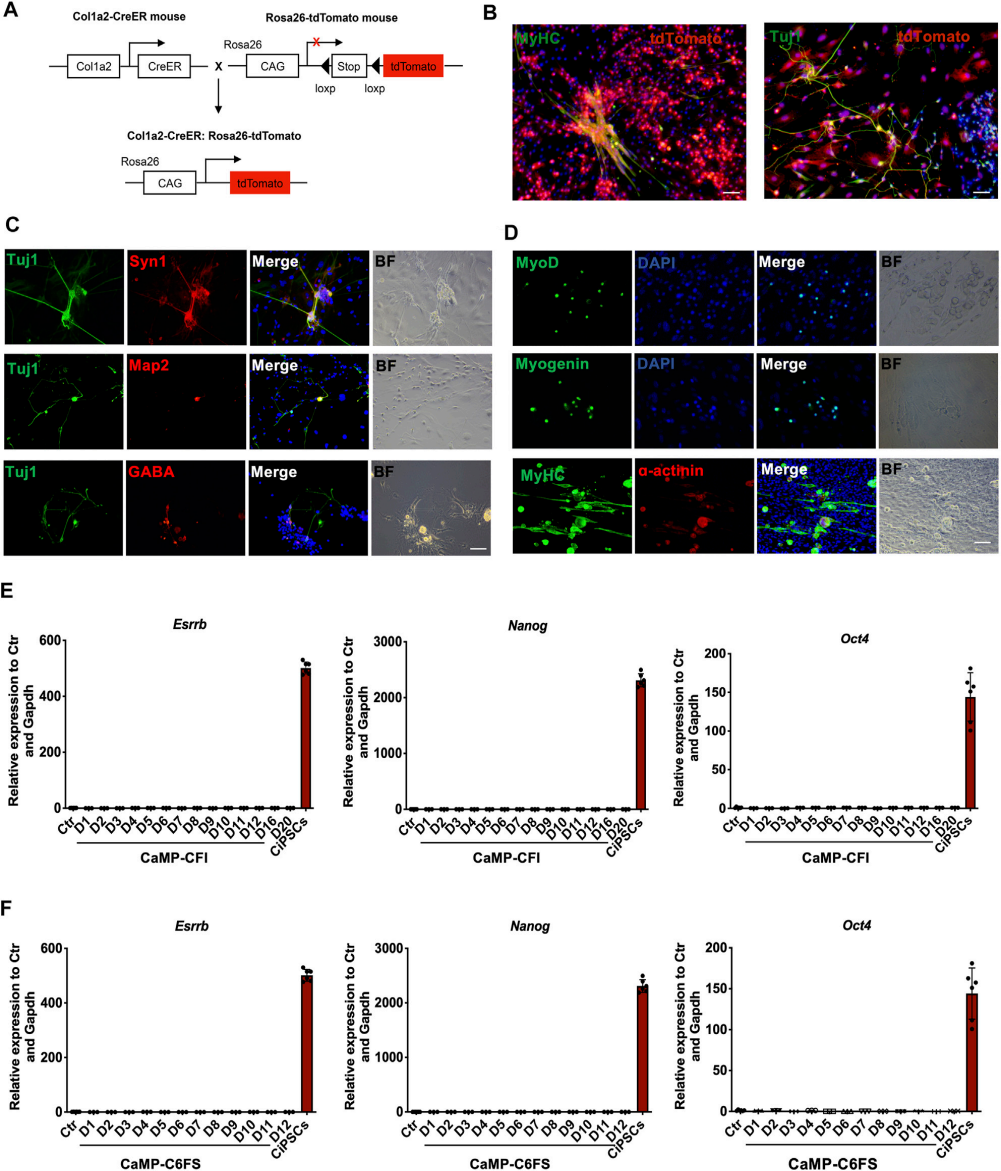
Cells induced from the CaMP state are not derived from progenitor cells or pluripotent stem cells. **(A)** Schematic diagram of Col1a2-derived lineage-tracing system. **(B)** Immunofluorescence for Tuj1-positive neuron-like cells and MyHC-positive myocytes that induced from Col1a2-positive fibroblasts. **(C)** Immunostaining for neuron-like cells induced from TTFs with CaMP pretreatment. Scale bar, 100 μm. **(D)** Immunostaining for skeletal muscle cells induced from TTFs with CaMP pretreatment. Scale bar, 100 μm. **(E)** Relative mRNA expression of hallmark pluripotent genes (*Nanog*, *Oct4*, and *Esrrb*) through the neuron-like cells induction process. **(F)** Relative mRNA expression of hallmark pluripotent genes (Nanog, *Oct4*, and *Esrrb*) through the skeletal muscle cells induction process. Data are presented as mean ± SD, ***p < 0.001; **p < 0.01; *p < 0.05, *t*-test.

To determine whether the induced neuron-like cells and myocytes were derived from fibroblasts and to rule out the contamination of progenitor cells in fibroblast culture, we applied the Col1a2 lineage-tracing system during the induction of neuron-like cells and myocytes. By immunostaining, we found that Tuj1 or MyHC expressing cells were mostly induced from Col1a2 expressing cells, suggesting that neuron-like cells were induced from fibroblasts rather than contaminated progenitors (Figures 7A,B). Furthermore, we induced adult mouse tail tip fibroblasts into neuron-like cells and beating myocytes expressing specific markers by introducing CaMP state (Figures 7C,D), which confirmed that CaMP induced plasticity in TTFs and further ruled out the contamination of neural progenitor cells in MEFs.

Besides, we had not detected the endogenous expression of pluripotent genes, such as *Nanog*, *Esrrb*, and *Oct4*, throughout the chemical reprogramming processes by RT-qPCR analysis (Figures 7E,F). The Oct4-GFP was not activated during the chemical reprogramming processes to neuron-like cells and myocytes by daily observation. These indicated that the neuron-like cells induction processes initiated by CaMP pretreatment did not activate the pluripotent genes.

**FIGURE 8:**
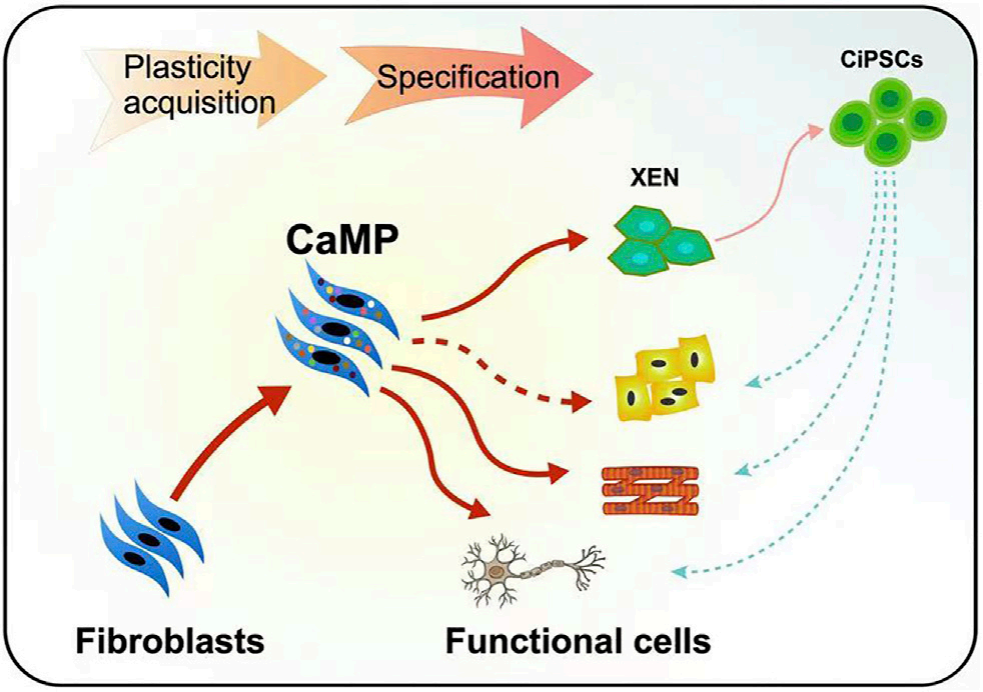
Graphical abstract of the plastic CaMP state amenable to direct lineage reprogramming.

Moreover, we found that the neuron-like cells and myocytes induced through the CaMP phase did not require the intermediate XEN-like states. The gene *Sall4*, a typically expressed master TF of XEN cells, had low expression in the process of reprogramming. The knockdown of XEN master genes, *Sall4* and *Gata4*, impaired the formation of XEN-like colonies but did not decrease the induction efficiency of neuron-like cells or myocytes (Supplementary Figure S3). It indicated a more direct cell fate conversion from fibroblasts to target cell types without establishing the XEN-like cell identity (Figure 8). These findings supported that cell plasticity with neuron-like cells and myocyte lineage specification potential was induced during the CaMP process.

## Discussion

In this study, we found that the cell fate specification was not initially determined in the chemical reprogramming process. Instead, a plastic CaMP state was induced, with the heterogeneous expression of multiple developmental genes, and without the determination to any specific cell fate. The establishment of such cell plasticity may account for the common roles of these key chemicals used in the CaMP induction in inducing different cell types.

Our findings provide a new understanding of cell plasticity and stability. Although it has been reported that the master regulators of cell fates, such as tumorigenicity-related genes, are always regulated strictly by multiple epigenetic mechanisms (Graf and Enver, 2009; Li et al., 2016; Dhar et al., 2018), our findings suggest that a considerable number of developmental-associated TFs are not quite strictly regulated in fibroblasts. These suggest that somatic cells possess plasticity in response to exogenous stimuli, in terms of expressing master genes for another cell fate, which could be an initial step and a priming phase for cell fate conversion (Dhar et al., 2018). In addition, the CaMP state would be reminiscent of pluripotent stem cells (PSCs) regarding their specification potential into cells of differentiated germ layers, such as myocytes and neuron-like cells as indicated in this study, as well as extraembryonic cell types like XEN-like cells in the previous report (Zhao et al., 2015). Even PSCs have a priming state with stochastically low expression of developmental genes (Bernstein et al., 2006), similar to the stochastic activation of developmental genes in the CaMP cells revealed in this study. These may suggest similar molecular bases for cell plasticity in the CaMP phase and pluripotent cells. In contrast, cells in the CaMP phase differ from pluripotent stem cells regarding their different gene expression profiles, spontaneous differentiation potential, and development potential in a single cell.

Furthermore, cells in the CaMP phase may not have the potential to differentiate by nature, with the fact that the treatment of chemicals and culture medium after CaMP are also essential for determining the cell fate specification derivate. Our previous study found that the trigger for cell fate specification is also very critical to hierarchically activating all the essential TFs for cell fate determination and transition (Yang et al., 2020). The initial fibroblasts program could not be substantially impaired unless major master TFs for another cell type are all co-expressed in the final transition stage (Yang et al., 2020). These support the concept that cell fate is somehow stable and not easily reversible, although easily primed.

In comparison, the process of transgenes *OSKM*- (*Oct4*, *Sox2*, *Klf4*, and *c-Myc*) induced pluripotent stem cell (iPS) generation is also a similar biphasic process, with an early stochastic gene-activation stage induced mainly by *c-Myc* and a late, more determined process mainly orchestrated by *OSK*, downstream of *Sox2* expression (Sridharan et al., 2009; Buganim et al., 2012; Polo et al., 2012). Recently, the heterogeneity of early-reprogramming cells expressing considerable development-associated genes induced by OSKM has also been reported (Schiebinger et al., 2019). These suggest that the biphasic “plasticization and specification” process revealed in our study could be a general principle for cell-fate reprogramming for both chemical and transgenic reprogramming.

Importantly, by harnessing the CaMP state induced in the initial stage of chemical reprogramming, we improved the reprogramming systems towards neuron-like cells and myocytes with pure chemicals by pretreating the cells with the CaMP inducing chemical cocktails. Moreover, the resting membrane potential of CaMP-induced neuron-like cells was –48.68 ± 2.43 mV (Supplementary Table S1), which was significantly lower than other reported chemical-induced neurons (–25 or –35 mV). It indicates that CaMP-induced neuron-like cells had more complete ion channels and were functionally closer to primary neurons. Besides, rather than using limited genes as biomarkers (typically done in other reported chemical reprogramming systems) (Hu et al., 2015; Li et al., 2015; Li et al., 2017), we compared all differentially expressed genes among samples. We found that CaMP-induced neuron-like cells had more similar transcriptional profiles to the primary functional cells. Overall, the CaMP-induced neuron-like cells were more mature in transcriptional profile and function than those previously reported. It would also be interesting to further determine whether chemical reprogramming through the CaMP state can be extended to obtain other functional cell types as a general strategy for developing chemical reprogramming systems and even be applied to human cells.

Similar to this strategy, a cell activation and signaling-directed (CASD) strategy, has been reported by transiently overexpressing Yamanaka factors, *OSKM*, to obtain different functional cell types, such as hepatocytes, pancreatic beta cells, and cardiomyocytes (Efe et al., 2011; Li et al., 2014; Wang et al., 2014; Zhu et al., 2014), although it was found that this strategy involved the transient acquisition of pluripotency (Bar-Nur et al., 2015; Maza et al., 2015). However, in chemical reprogramming through the CaMP state, it is not likely that chemicals induced a pluripotent state in the initial first 4 days of the 40 days long chemical reprogramming process towards CiPSCs. The chemicals rather established a more plastic state with a more active epigenetic state beneficial for transcriptional activation. Besides, it has been reported that the XEN-like cells induced during chemical reprogramming to CiPSCs are also plastic and can be further induced into other cell lineages, such as neurons or hepatocyte-like cells (Li et al., 2017). In comparison, our study showed that cell plasticity can be induced at the very beginning of chemical reprogramming for further cell specification, even before the establishment of XEN cell identity and without substantial silencing of core transcriptional networks of fibroblasts.

In comparison with cell differentiation from induced pluripotent stem cells or expandable XEN-like cells, cell fate lineage reprogramming systems through the CaMP state are more direct, bypassing the concerns of potential tumorigenicity resulting from uncontrolled cell expansion in *in vivo* applications and has the potential to be induced to cell types of all three germ layers. As a result, direct cell fate reprogramming through the CaMP state may be a new paradigm and a shortcut to obtaining functional cells for regenerative medicine (Figure 8).

## Conclusion

This study enlightens the understanding of chemical reprogramming by dissecting the contribution of reprogramming chemicals to the activation of development-associated transcription factors. It proves a new approach to obtain functional cell types through a CaMP state in the future of regenerative medicine.

## Data Availability

The datasets presented in this study can be found in online repositories. The names of the repository/repositories and accession number(s) can be found below: https://www.ncbi.nlm.nih.gov/geo/, GSE155818.
