# Supplementary figures and images for: Chemical Pretreatment Activated a Plastic State Amenable to Direct Lineage Reprogramming
Source: Front Cell Dev Biol. 2022 Mar 25;10:865038. doi: 10.3389/fcell.2022.865038 (PMC8990889; doi:10.3389/fcell.2022.865038)

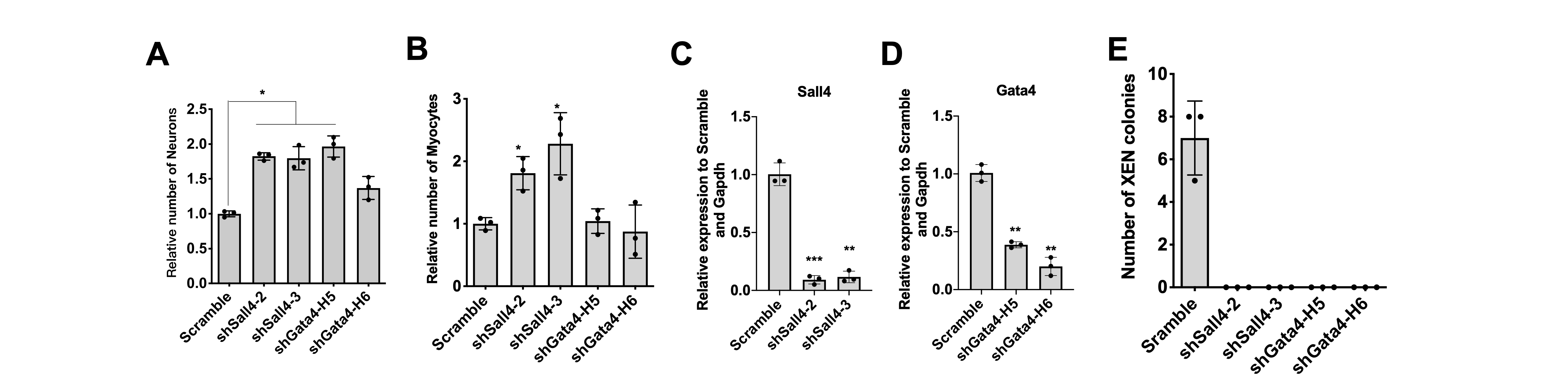

Supplement: Supplementary file 2 [file Image3.JPEG]

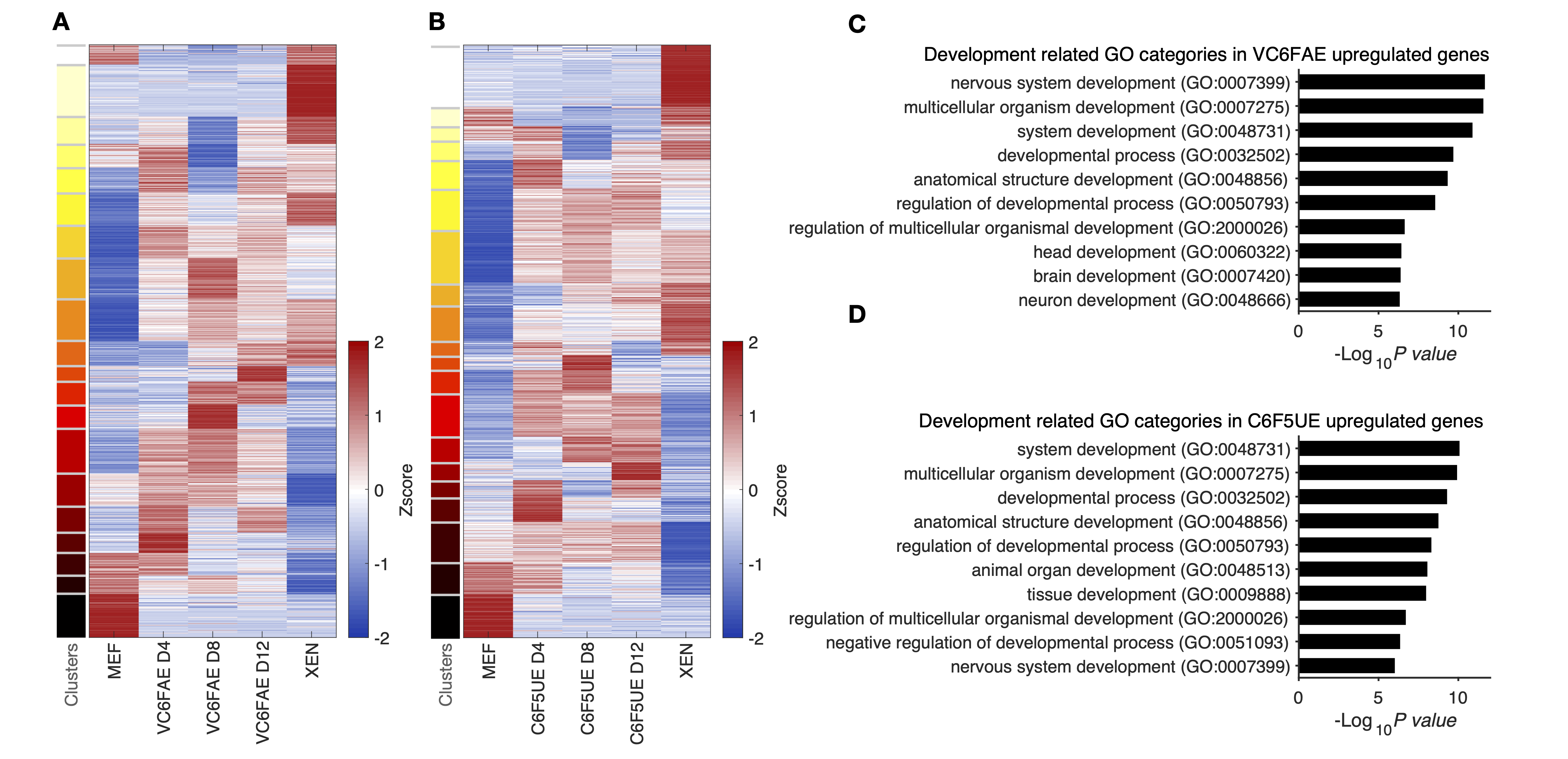

Supplement: Supplementary file 3 [file Image1.JPEG]

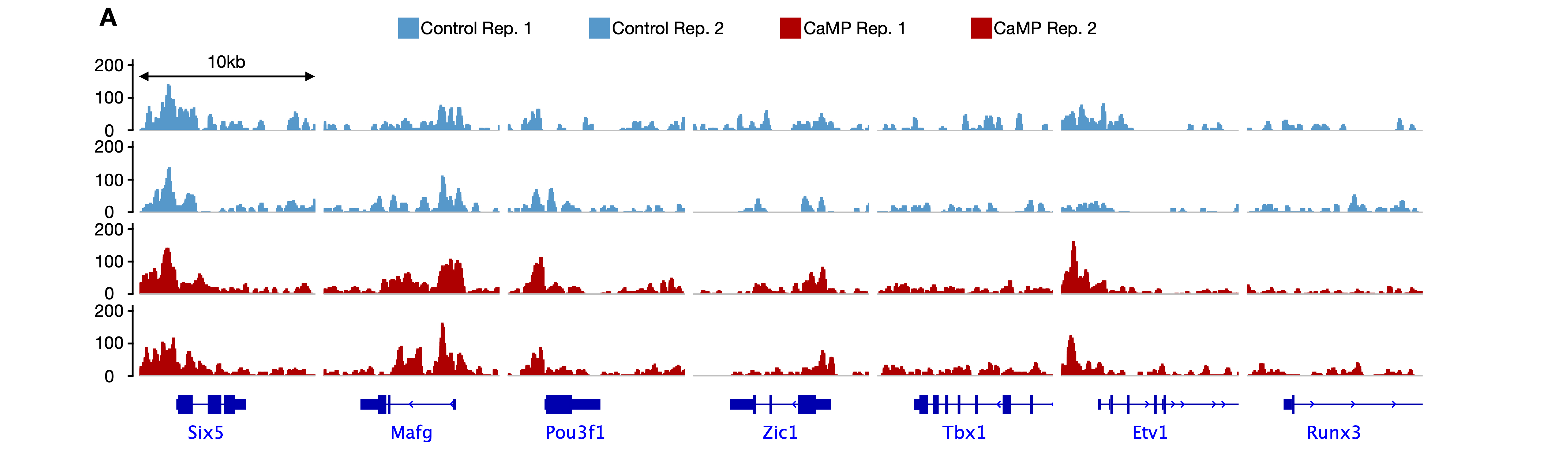

Supplement: Supplementary file 4 [file Image2.JPEG]
