# Supplementary material for: Chemical Pretreatment Activated a Plastic State Amenable to Direct Lineage Reprogramming
Source: Front Cell Dev Biol. 2022 Mar 25;10:865038. doi: 10.3389/fcell.2022.865038 (PMC8990889; doi:10.3389/fcell.2022.865038)
Supplement: Supplementary file 7 [file DataSheet1.docx]

**Supplementary legends**

**Supplementary Figure S1**

(A, B). Clustering of temporal gene expression dynamics in the early stage of chemical reprogramming with cocktails of VC6FAE and C6F5UE. *K*-means clustering partitioned genes which FPKM > 1 at least one time point into 20 clusters.

(C, D). Gene ontology analysis of activated endogenous TFs induced by VC6FAE and C6F5UE.

**Supplementary Figure S2**

(A). ATAC signal visualization of representative TFs which have high level chromatin accessibility both in Control and CaMP samples.

**Supplementary Figure S3**

(A, B). The relative number of Tuj1^+^ neuron-like cells and MyHC^+^ skeletal muscle cells after *Sall4* and *Gata4* knockdown (analyzed on day 16).

(C, D). *Gata4* and *Sall4* mRNA expression levels when *Gata4* and *Sall4* were knockdown, respectively. (analyzed on day 16 by RT-qPCR).

(E). The number of XEN colonies induced with *Sall4* or *Gata4* knockdown before passage at day 16.

**Data are presented as mean ± SD, ***p < 0.001; **p < 0.01; *p < 0.05, t test.**

**Supplemental table 1.** Electrophysiological properties of neuron-like cells induced from CaMP state.

**Supplemental Video 1**. Contractile skeletal muscle cells induced by CaMP-C6FS on day 12.
